# Draft Genome Sequence of the Anoxygenic Phototrophic Bacterium *Rhodoferax* sp. Strain U11-2br, Isolated from a Mountain Lake on the Ulagan Plateau

**DOI:** 10.1128/MRA.00675-21

**Published:** 2021-10-14

**Authors:** E. D. Bakhmutova, A. O. Izotova, S. V. Toshchakov, Z. B. Namsaraev, N. I. Yermolaeva, A. V. Komova

**Affiliations:** a National Research Center “Kurchatov Institute”, Moscow, Russia; b Institute for Water and Environmental Problems, Siberian Branch of the Russian Academy of Sciences, Barnaul, Russia; University of Maryland School of Medicine

## Abstract

We report the draft genome sequence of an anoxygenic phototrophic bacterium, *Rhodoferax* sp. strain U11-2br, which was isolated from a freshwater mountain lake on the Ulagan Plateau (Altai, Russia). The assembly contains 4,514,979 bp, with a GC content of 59.9%.

## ANNOUNCEMENT

The Ulagan Plateau is located in the south of Western Siberia. Research on the area has been mainly focused on paleoecology (1) and archaeology (2). Here, we present the first results of a microbiological study of the mountain lakes of the Ulagan Plateau.

*Rhodoferax* sp. strain U11-2br was isolated from a small (0.01-km^3^) freshwater mountain lake (50°24′32″N, 87°35′60″E). The lake is surrounded by a mountain coniferous forest. At the time of sampling (July 2018), the water temperature was 12°C and the total mineralization was 0.098 g/liter (pH 8.44). A combined sample of sediment and water column was used to obtain an enrichment culture in the light. The U4 medium for the enrichment culture and subsequent agar shake dilutions contained the following: NaHCO_3_, 115 mg/liter of distilled water; KNO_3_, 0.5 mg/liter; Na_2_SO_4_, 6 mg/liter; NaCl, 1.5 mg/liter; K_2_HPO_4_, 10 mg/liter; sodium acetate, 500 mg/liter; the pH of the medium was adjusted to 8.5. Light-brown colonies composed of slightly curved motile rods were developed in an agar shake tube, and a pure culture was obtained by separating a single colony. The pure isolate was confirmed using optical microscopy and 16S rRNA gene sequencing, and it was designated *Rhodoferax* sp. strain U11-2br.

Genomic DNA was isolated from the cell culture using the QIAamp DNA minikit (Qiagen, Dusseldorf, Germany) following the manufacturer’s recommendations. A paired-end DNA library (average insert size, 310 bp) was constructed using the Nextera XT DNA library preparation kit for Illumina (Illumina, USA). The DNA library was sequenced using an Illumina MiSeq system with 150-bp paired-end reads.

The draft genome sequence of *Rhodoferax* sp. strain U11-2br was constructed using the Shovill v. 1.1.0 pipeline (3), with read trimming using Trimmomatic v. 0.39 (4). Error correction of the Illumina reads was conducted using Lighter v. 1.1.2 (5). Overlapping and stitching of paired-end reads were completed with FLASH v. 1.2.11 (6). These reads were assembled *de novo* with SPAdes v. 3.14.1 (7). As a result, 78 contigs were assembled (*N*_50_, 108,245 bp). The draft genome sequence was annotated with the NCBI Prokaryotic Genome Annotation Pipeline (PGAP) (8). The genome size is estimated to be 4,514,979 bp, with a GC content of 59.9%. Default parameters were used except where otherwise noted.

The sequenced genome was compared to the closest neighbors’ genomes with the ChunLab online average nucleotide identity (ANI) calculator (https://www.ezbiocloud.net/tools/ani), which uses the OrthoANIu algorithm (9). ANI analyses confirmed discrepancies with the genomes of the closest neighbors.

The *Rhodoferax* sp. strain U11-2br genome has an ANI value of 75.64%, compared with the genome of Rhodoferax ferrireducens T118 (GenBank accession number GCA_000013605.1), with an average aligned length of 1,246,512 bp. The *Rhodoferax* sp. strain U11-2br genome has an ANI value of 93.55%, compared with the genome of Rhodoferax fermentans DSM 10138 (GenBank accession number GCA_016583655.1), with an average aligned length of 2,631,570 bp.

The draft genome sequence contains 4,101 coding sequences in total, 9 rRNAs (including three 5S rRNAs, three 16S rRNAs, and three 23S rRNAs), 49 tRNAs, and 3 noncoding RNAs. The draft genome sequence of *Rhodoferax* sp. strain U11-2br contains genes responsible for the metabolism of aromatic compounds (protocatechuate 4,5-dioxygenase subunit alpha [accession number MBT3067934.1], protocatechuate 3,4-dioxygenase [accession number MBT3067933.1], 4-carboxy-4-hydroxy-2-oxoadipate aldolase/oxaloacetate decarboxylase [accession number MBT3067937.1], LysR family transcriptional regulator [accession number MBT3067941.1], 4-hydroxybenzoate 3-monooxygenase [accession number MBT3067939.1], benzoate/H(+) symporter BenE family transporter [accession numbers MBT3066246.1 and MBT3069093.1], and others). In addition, genes involved in detoxification of a variety of compounds (chromate efflux transporter [accession number MBT3066395.1], chromate transporter [accession number MBT3065318.1] and others) were detected.

### Data availability.

The genome has been deposited in NCBI GenBank and is available under accession number JAHFZF000000000.1. Raw sequencing reads are available in the NCBI SRA under accession number SRR14650906.
